# The Melatonin–Mitochondrial Axis: Engaging the Repercussions of Ultraviolet Radiation Photoaging on the Skin’s Circadian Rhythm

**DOI:** 10.3390/antiox12051000

**Published:** 2023-04-26

**Authors:** Ting Gao, Yixuan Li, Xiaoyu Wang, Fazheng Ren

**Affiliations:** Key Laboratory of Precision Nutrition and Food Quality, Key Laboratory of Functional Dairy, Ministry of Education, Beijing Laboratory of Food Quality and Safety, Department of Nutrition and Health, China Agricultural University, Beijing 100083, China

**Keywords:** ultraviolet radiation, circadian rhythm, melatonin, skin photoaging, mitochondria, oxidative stress

## Abstract

Sunlight is a vital element in modulating the central circadian rhythm, such as the regulation of the host’s sleep–awake state. Sunlight is also considered to have a significant influence on the circadian rhythm of the skin. Over-exposure or prolonged exposure to sunlight can lead to skin photodamage, including hyperpigmentation, collagen degradation, fibrosis, and even skin cancer. Thus, this review will focus on the adverse effects of sunlight on the skin, not only in terms of photoaging but also its effect on the skin’s circadian rhythm. Mitochondrial melatonin, regarded as a beneficial anti-aging substance for the skin, follows a circadian rhythm and exhibits a powerful anti-oxidative capacity, which has been shown to be associated with skin function. Thus, the review will focus on the influence of sunlight on skin status, not only in terms of ultraviolet radiation (UVR)-induced oxidative stress but also its mediation of circadian rhythms regulating skin homeostasis. In addition, this article will address issues regarding how best to unleash the biological potential of melatonin. These findings about the circadian rhythms of the skin have broadened the horizon of a whole new dimension in our comprehension of the molecular mechanisms of the skin and are likely to help pharmaceutical companies to develop more effective products that not only inhibit photoaging but keep valid and relevant throughout the day in future.

## 1. Introduction

Aging is a complex, multi-factorial process and is characterized by a decline in the functions and capabilities of all tissues and organs and increased possibilities of disease and death due to a synergistic combination of intrinsic and extrinsic effects [1]. As the world’s demographics change, clinicians are faced with a growing population of older adults; by 2030, the elderly population is expected to increase to 71 million [2]. The population aged ≥ 65 is predicted to raise from 53 million in 2018 to 88 million in 2050 in the US [3]. The fiscal burden of age-associated health disorders will also increase in response to the increase in the elderly population. The anti-aging product market is predicted to grow to over 300 billion by 2021 [4]. As a result, there is a growing interest in counteracting biological processes.

The skin is the organ that changes most significantly during the aging process, with significant alterations in the structure and function of the skin. It is hypothesized that more than 80% of older adults suffer from different types of skin conditions, including life-threatening diseases (such as squamous cell carcinoma, basal cell carcinoma, and melanoma) to more common conditions (anemic eczema) [5]. As the largest organ in the host and covering approximately 95% of the host, skin is the first physical and immunologic barrier to the extrinsic environment and plays important roles in protection from external threats, regulation of the body temperature, sensation, secretion, excretion, and immunity [6,7,8]. Skin aging is the result of a combination of endogenous and exogenous aging; the former is dependent on aging, and the latter includes temperature, humidity, wind, ultraviolet radiation (UVR), etc. [9]. Overexposure to UVR is a vital factor among the external factors that induce skin photoaging, which manifests as the deposition of wrinkles, discoloration, telangiectasias, and dry and roughed skin [10,11]. These are closely related to the pathophysiological alterations in different cells and tissues in the epidermis and dermis. For example, wrinkles, as the most significant cosmetic change resulting from photoaging, are mainly caused by a reduction in dermal fibroblasts and a slower synthesis and faster breakdown of collagen and elastin [12]. Skin photoaging not only influences the aesthetics of the skin but also disrupts the structure and function of the healthy skin barrier, increasing the risk of skin inflammation and even malignancies [13]. Therefore, an in-depth molecular understanding of human skin photoaging caused by UVR is of scientific and clinical importance.

Thus, this review will focus on the influence of sunlight on skin status, not only in terms of UVR-induced oxidative stress but also its mediation of circadian rhythms regulating skin homeostasis. In addition, this article will address issues regarding how best to unleash the biological potential of melatonin. These findings about the circadian rhythms of the skin have broadened the horizon of a whole new dimension in our comprehension of the molecular mechanisms of the skin and are likely to help pharmaceutical companies to develop more effective products that not only inhibit photoaging but also remain valid and relevant in the future.

## 2. Mechanism of UVR-Induced Skin Damage

UVR can be divided into three types depending on different wavelengths: UVA (320–400 nm), UVB (280–320 nm) and UVC (100–280 nm) [14]. Changes in the structure and function of the skin induced by differential wavelengths of UV light vary significantly. Of these, UVC is totally absorbed by the ozone layer, and both UVA and UVB are vital contributors to skin diseases [15]. UVB can only penetrate the epidermis, while UVA accounts for 90–95% of the total UV and has strong penetrating power. UVA penetrates the papillary layer of the dermis and influences the cellular components of the dermis and even subcutaneous tissue areas, e.g., fibroblasts, vascular endothelial cells, and Langerhans cells, and activates matrix metalloproteinases (MMPs), accelerating the collagen degradation (type I and type III collagen) and elastic fibers, leading to the disruption of the dermal structure. This damage can have far-reaching effects on the dermal tissue, leading to skin laxity, sagging, abnormal increases in wrinkles, and other macroscopic photoaging injuries [16].

Currently, there are a number of mechanisms through which UVR induces skin photoaging, and in what follows, the most widely recognized processes are presented.

### 2.1. UVR-Mediated DNA Damage-Induced Skin Photoaging

High-energy UVB radiation (280–320 nm) penetrates the skin deep into the epidermis and causes severe epidermal injury, such as direct damage to DNA (e.g., cyclobutane pyrimidine dimer (CPD), pyrimidine (6–4) pyrimidine photoproducts (6-4PP), double-strand breaks (DSBs) and others) [17]. In addition, it creates an oxidizing environment that can result in mutagenic and carcinogenic effects [18]. The skin, located in the outermost layer of the host and exposed to harmful effects of the environment, including UVR, has evolved sophisticated mechanisms for local protection or recovery (skin) or to influence the balance of the whole body [19]. While causing DNA injury, UV irradiation also activates p53 oncogenic agents, synchronizes cellular responses, and leads to cell cycle arrest, which is vital for DNA repair or apoptosis; this allows for the organized elimination of potentially cancer-causing cells [20].

### 2.2. UVR-Mediated Overproduction of ROS-Induced Oxidative Stress and Apoptosis Lead to Skin Photoaging

UVA irradiation exposure may cause the overexpression of ROS levels in the skin, which could lead to oxidative stress and disrupt nucleic acids, proteins, and lipids [17]. UVA can produce ROS directly by affecting the cell cytoplasm and membrane or indirectly by causing mitochondria damage. Differences in the timing and sources of ROS generation also have different influences on cells [21]. The excess of ROS generation can disturb redox balance and lead to DNA injury via the production of cyclobutane pyrimidine dimers (CPDs), pyrimidine–pyrimidone (6–4) photoproducts, and 8-oxo-2′-deoxyguanosine (8-oxodG) [22], thus resulting in cell apoptotic effects, inflammatory response, and immunosuppression. Oxidative stress induced by the overproduction of ROS could change p53 folding and functions, further inducing UV-caused DNA injury and/or apoptotic response, which destabilizes the genome and allows malignant transformations to occur during the cell cycle [22].

### 2.3. UVR-Mediated Overproduction of Pro-Inflammatory Factors Induces Skin Photoaging

UVR promotes the overexpression of pro-inflammatory factors. Inflammatory factors exert an important effect on skin photoaging [23], and they are generated from different types of skin cells, such as fibroblasts, keratinocytes, and vascular endothelial cells, including plasma mediators, lipid mediators, and inflammatory cytokines [24]. ROS-mediated COX-2 and prostaglandin E2 (PGE2) are related to the upregulation of ornithine decarboxylase and promote cell migration [25,26,27]. UVR promotes the activation of subcutaneous inflammation factors that produce ROS, reactive nitrogen, and hydrogen peroxide, resulting in DNA deletion and reorganization [28,29]. UVR changes the distribution patterns of TGF-β, which induces extracellular matrix (ECM) remodeling by increasing MMPs and ultimately promoting skin photoaging [30].

### 2.4. UVR-Mediated Dysregulation of Immune Effects Induces Skin Photoaging

UVR not only decreases cell immunity but also affects humoral immunity [31]. UVR stimulates Langerhans cells in the epidermal, which are regarded as a vital factor in cellular immune responses associated with antigen delivery [32]. UVR not only decreases the number of Langerhans cells but also disturbs their efficacy, including lymphocyte activity and surface antigen content. Long-term UVR exposure decreases the level of stimulating molecules on the Langerhans cells, suppressing the synthesis of the membrane-related antigens B7 (B7-1, B7-2) [33]. Further, UVR increases macrophage contents in the epidermal layer, promotes regulatory T cells (Tregs), disrupts the immune homeostasis of T helper 1/2 cells (Th1/ Th2), and polarizes the Th1/ Th2 response to Th2 [34]. The immunosuppressive action of Th2 response polarization may rely on IL-12, resulting from IL-12 consumption that induces T-cell activation in favor of Th2 and promotes Treg activation [35,36,37].

## 3. Visible Light-Mediated Circadian Rhythm Regulates Skin Function

Most of the causative factors of skin photoaging caused by UVR are limited and do not explain the underlying mechanism of its occurrence. Perhaps we should consider the real causative factors of skin damage caused by UVR in terms of the overall effects of UVR on the host.

Over the past decade, research investigations have slowly begun to shift, not only focusing on UVR-caused skin photodamage but also including the effects of sunlight on the circadian rhythm of the skin. This is due to the fact that the skin also exhibits circadian rhythms, in which different levels of different types of skin cells have autonomous oscillations that are fine-tuned to cooperate with environmental alterations; this surpasses the common concept of human skin damage and regeneration, namely that it mainly acts as the first barrier against UVR attacks [38]. As a way to beneficially protect the host from UVR radiation, the function of skin cells relies on the host’s central circadian rhythm. Thus, it is important to explore the effect of sunlight on the host’s circadian rhythm.

**Figure 1:**
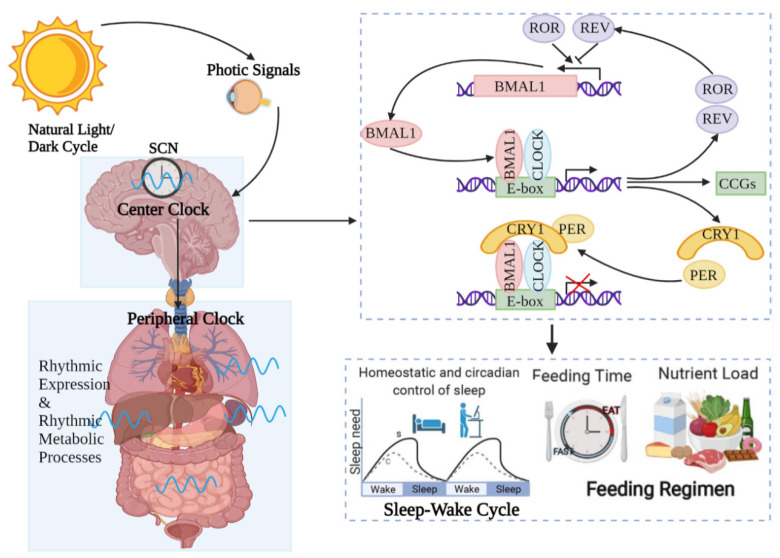
**Circadian rhythm regulates host activities.** Circadian rhythms are controlled by central modulators placed in the anterior–superior chiasmatic nucleus (SCN) of the hypothalamus. When the retina receives light, the central clock synchronizes the peripheral clock placed in tissues such as the skin through hormone and neuronal signals. Within the core loop, the heterodimer formed by CLOCK and BMAL1 proteins acts as a transcription factor that enhances the transcription of genes containing E-box-enhancer elements. These include genes that are important for negative feedback proteins PER and CRE. They are first translated and accumulated in the cytoplasm. After the formation of PER–CRY heterodimers, they translocate into the nucleus and inhibit the CLOCK–BMAL1 enhancer complex. As a result, they suppress their own transcription. The 24 h cycle is completed by controlling point-directed degradation of the PER–CRY complex by the proteasome. To control the periodic appearance of the CLOCK–BMAL1 enhancer complex, stable loops that express inhibitory REV-ERBa/b and enhance RORa/b are exchanged, thus regulating the coexpression of BMAL1 by competing for binding to the transcriptional enhancer site RORE on the BMAL1 promoter. As a consequence, circadian rhythms regulate almost all activities of the organism, such as the sleep–wake cycle and feeding time, etc.

On the one hand, UVR, as a visible light, regulates the circadian rhythms of the central and peripheral organs of the body, including the skin. Light is a vital environmental element that impacts life systems on Earth. It is not only an energy source for photosynthesis but also a message cue for the growth and development of photosynthetic organisms. The light–dark cycle is one of the most regularly cyclical signals of living organisms, regulating rhythmic alterations in the metabolism, physiology and behavior of most organisms (Figure 1).

On the other hand, although sunlight can play a positive and coordinated role in the growth and development of life on Earth, its harmful effects on living organisms are inevitable. Overexposure to UVR can cause serious damage to the human skin, resulting in erythema, photoaging, inflammation, and even cancer.

Although this may seem contradictory, the underlying mechanisms behind the dual role of sunlight need to be explored to find solutions that can both maintain the circadian rhythm of the skin and protect it from UVR-mediated skin photoaging.

### 3.1. Visible Light-Mediated Circadian Rhythms in Skin

The circadian rhythm is a 24 h cycle of physiological activity that is followed by the behavior and physiology of almost all organisms on Earth. When the retina captures light, the central modulator, situated in the anterior–superior hypothalamic nucleus (SCN), promotes and standardizes the circadian rhythms of peripheral organs through neuronal and endocrine signals [39]. At the molecular scale, mammalian circadian rhythms are controlled via the expression of core clock genes, including the circadian locomotor output kaput (CLOCK), brain and muscle ARNT-like protein 1 (BMAL1), periods (PER1, 2, and 3), and cryptochromes (CRY1 and 2). Using a transcription–translation feedback loop to modulate the clock mechanism, CLOCK and BMAL1 proteins originally compose a CLOCK–BMAL1 heterodimer that initiates the transcription of some genes with E-box cis-regulatory enhancer sequences, including the PER and CRY genes’ transcription. PER and CRY then compose PER–CRY heterodimers that inhibit the CLOCK–BMAL1 complex in the nucleus, negatively modulating its transcription [40].

**Figure 2:**
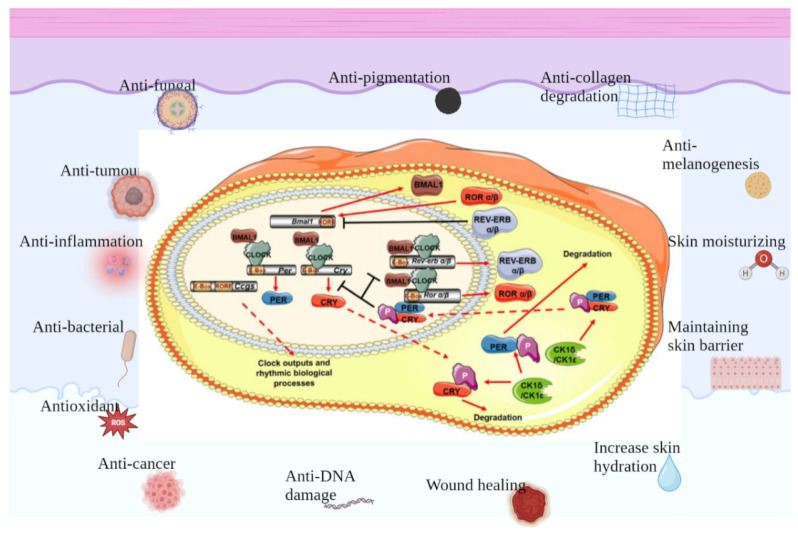
**Protective effects of clock genes on skin.** There is a separate set of biological clocks in the skin that regulate different functions of the different cells of the skin and work together to maintain skin homeostasis, which involves anti-fungal, anti-tumor, anti-inflammation, anti-bacterial, anti-oxidant and anti-cancer effects, as well as contributing to anti-DNA damage and wound healing, increasing skin hydration, maintaining skin barrier, moisturizing skin, anti-melanogenesis, anti-collagen degradation and anti-pigmentation.

In addition to modulating the genes of PER and CRY, CLOCK–BMAL1 promotes either the transcription of the retinoic-acid-associated orphan nuclear receptor (Rev-erbα) or retinoic-acid-related orphan receptor (Ror)α. Both serve as the regulators of BMAL1, promoting or suppressing its transcription by combining with retinoic-acid-associated orphan receptor response elements (ROREs) on the BMAL1 accelerator [40]. Moreover, it was also found that not only Rev-erbα and Rorα but also all members of Rev-erb (α and β) and Ror (α, β, and γ) regulate BMAL1 expression [41]. Advances in the study of clock genes and proteins have led to further comprehension of how circadian rhythms affect human biological processes, particularly through the light–dark cycle and the effects of sunlight (Figure 2).

### 3.2. The Skin Itself Also Exhibits Circadian Rhythms, Including Different Structures, Different Cells, and Different Physiological Indicators

Similar to other tissues and organs in the host, the skin also exhibits circadian rhythms. Skin functions, including transepidermal dehydration (TEWL), hydration, the modulation of skin pH and temperature, and barrier function, all exhibit circadian rhythms [42]. In the 24 h cycle, the intra-tissue daytime function is completely different from the nighttime function, requiring healthy skin by regulating energy delivery and cellular statuses for optimal protection and restoration. Circadian rhythms synchronize various skin cell types with each other and with the body’s natural rhythms.

#### 3.2.1. The Skin’s Circadian Rhythm Affects Drug Absorption, Permeability, and Blood Sample Rate

The influence that topical applications have on the skin also varies throughout the day. The skin penetration rate of hydrophilic and lipophilic topical applications is at its highest at about 04:00 h (4:00 a.m.), with absorption slowing throughout the daylight hours [43]. The penetration of topical lidocaine is likewise highest in the evening [44], which may be due to the increased permeability of the skin during evening hours. The blood flow rate is another factor that may impact the composition of topical drug escape and uptake [45]. Research indicated that the circadian rhythm’s blood flow rates were kept even during treatment with topical corticosteroids [46]. Vasodilation and increased skin blood flow have been proven to accelerate drug access through the skin into the systematic cycle [47].

#### 3.2.2. The Effect of the Skin’s Circadian Rhythm on Hair Follicle Growth

Host hair follicles also suffer circadian alterations and possess the core clock genes, namely CLOCK, BMAL1, and Per1, which regulate the hair follicle cycle [48]. A previous study suggests that these clock genes could serve as effective treatment targets for promoting hair growth [48]. Moreover, researches showed increased melanin content in hair follicles via the inhibition of the expression of BMAL1 and PER1, indicating a role of circadian clock genes in hyperpigmentation [49]. The interventions proposed for these genes may help treat hair pigmentation imbalance. Cortisol contents also ebb and flow throughout the whole day. Cortisol levels have a natural trough at night, which may be a trigger for patients with nocturnal pruritus [50]. About 65% of patients with allergic skin diseases have itching that worsens at night, including those with atopic dermatitis and psoriasis [51].

#### 3.2.3. The Skin’s Circadian Rhythm Affects the Skin Temperature and Pruritus in Psoriasis

Skin temperature and pruritus in psoriasis are also regulated by circadian rhythms [52]. There are slight fluctuations in core body temperature; daytime temperatures are slightly higher than nighttime, with a low point in the early morning, while skin temperatures peak in the afternoon and again decrease, reaching valley points at night [46]. Similarly, the pathogenesis of psoriasis is associated with a disturbance of circadian rhythms, which may be related to thermoregulation [53]. Psoriasis is also associated with abnormal circadian rhythms. A research study revealed an increased incidence of psoriasis in night-shift staff [54]. Moreover, the CLOCK gene is associated with the regulation of psoriasis by modulating the expression of interleukin-23R in mice [55]. Future research is needed to further elucidate the connection between psoriasis and the circadian clock.

#### 3.2.4. The Skin’s Circadian Rhythm Affects the Modulation of Epidermal Barrier Function

Since the epidermis has continuous cell turnover, proper timing is vital. The basal layer offers a regular supply of newly born cells to the epidermis. Stem or progenitor cells in the basal layer of the epidermis should divide once a day around the afternoon or early morning, pushing the cells above them to the outside of the epidermis. This process may cause keratinocyte proliferation concerning their present position in the spiny or granular layer and the stratum corneum, respectively. The circadian clock of keratinocytes relies on their position in the skin. The genes regulating keratinocyte differentiation are expressed late at night and early in the morning, implying that the recovery of the skin barrier begins in the morning, which is consistent with higher TEWL values detected in the morning than in the evening [56]. The genes regulating cell proliferation and differentiation in the basal lamina are expressed in the late afternoon and evening. At a macro level, this means that the skin barrier is strongest and the skin is most hydrated in the afternoon, while the skin barrier is least hydrated and strongest late at night/early in the morning [57]. This type of skin disease usually causes itchy skin that goes undetected during sleep.

#### 3.2.5. The Skin’s Circadian Rhythm Influences the Production of Inflammatory Factors

Among the predicted genes exhibiting rhythmic expression in the skin, the tissue suppressor of metalloproteinases (TIMP)3 is one of the first new CLOCK-dependent circadian genes identified by researchers, suggesting the value of its rhythmic expression under UVR conditions [58]. Researchers have shown that TIMP3 mRNA exhibits periodic oscillations in synchronized host keratinocytes, likely the core clock gene PER1 and the AQP3 gene regulating the epidermal circadian clock, but in the reverse phase to the core clock gene BMAL1, indicating that TIMP3 expression in the skin could be highest during the day and lowest at night. The reduction in the TIMP3 expression level under UVB irradiation and the concomitant increase in TNF-α production were restored after being supplied with synthetic TIMP3 peptides. The results indicate that TIMP3 exerts a positive influence as a protector against UV-caused cellular responses during the daytime in the host skin.

#### 3.2.6. The Skin’s Circadian Rhythm Affects the Expression of Water Channel Proteins

A previous study on the rhythmic alterations in aquaporin 3 (AQP3) and TIMP3 highlighted the significant role of circadian rhythm on the expression level of water channel proteins [59]. Due to the influence of UVB, the core clock genes, namely BMAL1 and CLOCK, were immediately reduced in NHEK cells. While CLOCK could resynchronize and return to the initial cadence mode, BMAL1 underwent a phase shift [58]. The reduction in clock genes was also accompanied by the inhibition of AQP3 and TIMP3 after exposure to UVB [59]. With the downregulation of TIMP3, TNF-α and MMP1 increased. In addition, the CCAAT-enhancer combining protein α (C/EBPα), chemokine (C-X-C motif ligand 1 (CXCL1), and IL-8 also increased with the inhibition of TIMP3 [58]. Moreover, the CLOCK gene knockout then exhibited a continuous suppression of TIMP3 rhythm mode, indicating that there is a correlation between TIMP3 synthesis with core clock genes [58]. It is also possible that PER3 is involved when cells are exposed to UVB. This is due to the inhibition of PER3, which is associated with an increase in MMP1 [60]. It is likely that after UVB exposure, the expression level of CLOCK decreases, influencing PER3 expression, and subsequently, TIMP3 is inhibited, allowing MMP1 to be uncontrollably upregulated.

In general, all biological functions in the host are adapted to a 24 h circadian cycle. In order for this biological rhythm (i.e., the circadian system) to function properly, all circadian clocks of the host need to be consistent with each other. To accomplish this task, a small number of neurons in the SCN in the brain function as central pacemakers for the entire body. Therefore, the circadian rhythm of the skin is also controlled by the circadian rhythm located in the SCN, and the influence of visible light on the circadian rhythm of the skin is also achieved by mediating the SCN’s circadian rhythm.

## 4. Melatonin, Circadian Rhythm, and Skin Homeostasis

Interestingly, the skin is an organ that secretes a variety of hormones that are essential for maintaining skin function. Several of these are also regulated by the modulator, such as circadian clocks and ROS. In addition, the skin itself can be considered a neuroendocrine type system that can be used not only for its own signaling but also to coordinate with the overall neuroendocrine system of the host [19,20,61]. The implication of this system is that the cells in the skin are capable of both producing and responding to the functions of conventional bioactive molecules [62]. The micromanagement of skin cells allows for supporting local balance as well as sending signals to the rest of the host to allow for comprehensive alternations [63].

Notably, the pineal hormone melatonin (N-acetyl-5-methoxytryptamine) has been proven to be crucial in keeping balance. The biologically active methoxyindole was initially regarded to be a factor in the maintenance of circadian rhythms, along with 5-hydroxytryptamine. Early studies on melatonin showed that it is a biomarker of circadian rhythms [64]. Melatonin levels are synchronized with circadian rhythms, with its levels peaking at night and having a trough during the day [65]. When exposed to UV light, melatonin levels sharply decrease due to feedback inhibition [50]. Meanwhile, UVR-mediated skin photoaging due to circadian rhythm disturbance results from excessive ROS accumulation, whereas melatonin inhibits UV-induced skin cell damage and exhibits a potent anti-oxidant effect in UV-exposed host cells, offsetting ROS production and protecting cells from mitochondrial and DNA injury [66]. Further, melatonin and its derivatives can also counteract the UVR-induced damage in human and porcine skin ex vivo [67].

**Figure 3:**
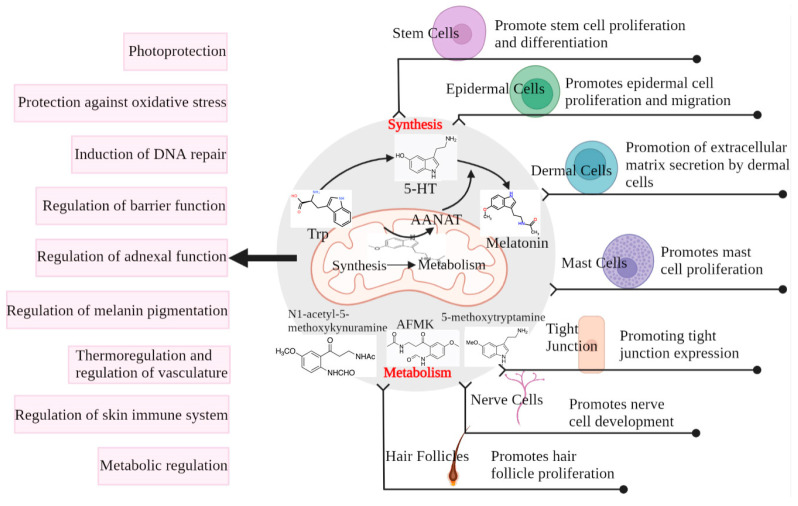
**Melatonin production and metabolism in the skin and its protective effect on the skin.** Cells use AANAT produced in the mitochondria from tryptophan to 5-HT for the eventual synthesis of melatonin, which can also be metabolized in the cells by metabolites such as AFMK. Both melatonin and its metabolites play a regulatory role in different structures of the skin, ultimately protecting the skin from damage [69].

Melatonin is associated with the modulation of a variety of skin functions, such as hair growth, the recovery of skin cells from UV damage, wound healing, and anti-tumor effects [51,68]. Considering the key role that melatonin exerts in the regulation of circadian rhythms and skin function, it is necessary to understand the properties of melatonin that allow it to intervene in skin photoaging (Figure 3).

### 4.1. Production and Metabolic Pathways of Melatonin in the Pineal Gland and Skin

Melatonin, a highly evolutionarily conserved molecule, has a variety of functional activities, such as the modulation of circadian rhythms and cellular responses required for cell survival and recovery of cellular balance [70,71,72,73,74,75]. Melatonin is not only produced in the pineal gland but is also found in some peripheral tissues and organs [60,61,62,63]. Similarly, melatonin is synthesized and metabolized in the skin [75,76].

Normal and damaged skin tissues not only secrete enzymatic processes but can also generate N-acetylserotonin, serotonin, and melatonin with their metabolites [76]. Recent studies have shown that melatonin may act as a double-edged sword when produced by malignant skin cells, e.g., protecting them from chemo- or radiotherapy [77]. The skin also has the ability to synthesize/recycle the (6R)-L-erythro-5,6,7,8-tetrahydrobiopterin, a cofactor for tryptophan hydroxylase (TPH) [78]. Hydroxytryptophan can also be nonenzymatically produced in the skin via H_2_O_2_ and the free-radical-induced oxidation of L-tryptophan caused by UVA exposure [79]. In addition to extensive TPH1 gene expression in skin cells [80], we also detected TPH2 in melanocytes, dermal fibroblasts [81], and the retinal pigment epithelium [82]. Skin could also express alternating splicing forms of the melatonin synthesis pathway: TPH, arylalkylamine N-acetyltransferase, and N-acetylserotonin O-methyltransferase [83]. Cutaneous N-acetylserotonin is expressed by both arylalkylamine N-acetyltransferase and arylamine N-acetyltransferase [84,85]. Melatonin in the skin is metabolized via indole, kynurate, and P450-dependent pathways or through UVR or free-radical-induced nonenzymatic processes [75,86,87,88].

Although immunocytochemistry has been used to identify N-acetylserotonin and melatonin antigens in epidermal and follicular keratinocytes and melanocytes, adnexal structures, fibroblasts, endothelial cells, and mast cells [80,89], it is only recently that researchers have been able to quantify melatonin and its metabolites in the human epidermis using mass spectrometry [90,91]. Epidermal melatonin production relies on gender, race, and age with the highest melatonin concentrations in African Americans. Among these metabolites, 6-hydroxymelatonin showed the highest levels, followed by 5-methoxytryptamine, *N*1-acetyl*N*2-formyl-5-methoxykynuramine (AFMK), and *N*1-acetyl-5-methoxykynuramine [90,91]. AFMK and *N*1-acetyl-5-methoxykynuramine levels were the highest in African Americans, and no obvious variance was observed for 6-hydroxymelatonin and 5-methoxytryptamine. However, dermatopathopathological-associated alterations in melatonin and its metabolites in the epidermis should continue to be systematically explored to dissect the relevancy between its endogenous expression and metabolic depletion and defined skin diseases.

### 4.2. Melatonin Exerts Photoprotective Skin Effects through Circadian Rhythm Regulation

Previous studies have shown that melatonin upregulates PER1 levels in healthy human keratinocytes, suggesting that it directly participates in regulating the skin’s circadian rhythm [92]. Regarding the 24 h light–dark cycle, melatonin is highest at night, and it affects the expression of the PER1 gene in the skin. It was found that when fibroblasts were supplied with melatonin 2 h after the peak of PER1, the amplitude of PER1 obviously increased by 28%. Thus, melatonin can regulate the amplitude of circadian rhythms in cultured fibroblasts. There are two hypotheses to explain this effect. Either melatonin increases the amplitude of the clock at the cellular concentration, or it gently resets the individual oscillators, increasing synchronization at the cultural level.

Moreover, the acetylation status of BMAL1 plays a vital role in the modulation of the circadian clock. The histone acetyltransferase (HAT) activity of CLOCK and the histone deacetylase (HDAC) activity of SIRT1 determine this acetylation status [93,94]. Studies have indicated that melatonin is a suppressor of SIRT1 HDAC activity and a suppressor of SIRT1 expression, suggesting that it has a vital role in managing circadian rhythms through its influences on SIRT1 [95]. Furthermore, it has been established that the addition of exogenous melatonin to prostate cancer cells can re-establish normal circadian oscillations [96]. Therefore, melatonin concentrations are critical when developing effective therapies for circadian rhythm disorders.

### 4.3. Melatonin Exerts Photoprotective Skin Effects through Anti-Oxidant Regulation

Melatonin is thought to protect the skin through different pathways, one of which is through its anti-oxidant benefits [97,98]. Melatonin, as an anti-oxidant, is a powerful scavenger of UV-caused ROS, suppressing potential DNA injury that could induce cancer. Melatonin has been found to be a more potent scavenger of ROS than vitamin C or vitamin E, which have been used to treat cytotoxic events [99].

#### 4.3.1. The Melatonin–Mitochondrial Axis Mediates Redox Homeostasis in Skin

It is known that ROS are produced in the mitochondria, which are also the site of melatonin synthesis. Therefore, the interaction between melatonin and the mitochondria plays an important role in ameliorating UVR-mediated skin photoaging induced by circadian rhythm disturbance. Notably, the new melatonin–mitochondrial axis has recently been regarded as a vital medium of epidermal balance, where melatonin and its metabolites coordinate the mitochondrial modulation of skin fate by influencing decisions between cell proliferation and differentiation, entry into terminal differentiation as part of the epidermal barrier structure, or escape from malignant transformation through apoptosis [75].

#### 4.3.2. Protective Effect of Melatonin on Mitochondria in Skin

The mitochondria have been shown to be one of the nonclassical targets of melatonin [100]. Melatonin suppresses UV-activated apoptosis response [101]. The suppression of UV-caused apoptosis was exhibited in the terminal deoxynucleotidyl-transferase-mediated (dUTP) nick-end labeling (TUNEL) test in host HaCaT keratinocytes [86]. Melatonin has also been found to suppress the production of cytochrome c in the mitochondria [102]. Moreover, melatonin was found to reduce the activation of Caspases 3, 7, and 9 in UV-irradiated keratinocytes, suggesting the suppression of the mitochondrial apoptotic pathway [99]. The protective effect of melatonin on the mitochondria not only depends on the direct clearing of ROS but also on the maintenance of an optimal mitochondrial membrane potential (Δψm) [102] and cell membrane pH [102]. Studies have shown that the protective influence of melatonin on the mitochondria is achieved through the direct or receptor-mediated suppression of the mitochondrial permeability transition pore (MPTP) and the stimulation of uncoupling proteins (UCPs) [100]. However, this speculation remains to be confirmed in skin cells. Recent studies have shown that the regulation of mitochondrial homeostasis is complex and concentration-dependent [103]. At nanomolar and sub-nanomolar concentrations, melatonin briefly activated the expression of nNOS via the receptors of MT1 and MT2, which leads to the NO-mediated regulation of mitochondrial function (suppression of the oxidative phosphorylation and reduction in the mitochondrial membrane potential and ATP synthesis). However, at higher levels (>1 nM), melatonin would come in contact with calmodulin, resulting in nNOS suppression [103]. Melatonin and its metabolites, including 6-OHM, AFMK, AMK, NAS, and 5-MTm upregulated the ability of host keratinocytes and melanocytes exposed to UVB irradiation [101]. Furthermore, an increased concentration of glutathione and reduced concentrations of nitrate and hydrogen peroxide were observed in keratinocytes supplied with melatonin metabolites and ultimately irradiated [104]. Thus, a recent study revealed that melatonin at a concentration of 1 mM offset the inhibitory effect of UVB on ATP production and decreased the ROS level in irradiated cells [105].

#### 4.3.3. Mechanism of Mitochondrial Protection by Melatonin in Skin Photoaging

What is the potential mechanism for melatonin accumulation in the mitochondria against a content gradient? Melatonin is a lipophilic substance that could easily traverse the plasma membrane. However, this does not explain the phenomenon of the passive diffusion of melatonin. Studies have suggested that melatonin can be transported into cells with the help of glucose transporter 1 (GLUT1) and that this function relies on the glucose content [106]. The transporter’s capabilities for the transfer of melatonin into the mitochondria remain unknown. However, recent studies indicated that peptide transporters 1 and 2 (PEPT1/2), also called the solute carrier family 15 members 1 and 2 (SLC15A1/2), were detected in the mitochondrial membrane, which is used for melatonin transport into the mitochondria. The concentration of these transporters in the mitochondria was positively associated with the level of mitochondrial melatonin. Melatonin may be transported to this organelle via PEPT1/2 located on the mitochondrial membrane. This active transport process leads to the accumulation of mitochondrial melatonin and provides cytoprotection [107]. Mansouri et al. indicated that melatonin ameliorated ethanol-induced mitochondrial DNA depletion in a mouse model through a mechanism associated with the anti-oxidant function of melatonin. Martin et al. further found that melatonin inhibited the suppression of mitochondrial complexes I and IV caused by ruthenium red and obviously decreased the mitochondrial oxidative stress induced by t-butyl hydroperoxide [108]; by contrast, comparable doses of vitamins C and E prevented these beneficial effects [109]. The relative protection of the mitochondria by melatonin differs from that of vitamin C/E, probably due to the observation that melatonin may accumulate in the mitochondria through active transport via PEPT1/2, which is responsible for melatonin’s protective effect compared with vitamin C and E.

Huo et al. also showed that the host’s oligopeptide transporter PEPT1/2 is associated with regulating the migration of melatonin into cells and, importantly for the present research, into the mitochondria [110]. Through several cancer cell models, the team probed melatonin as a mediator for the PEPT1/2 transporter. Extensive researches have demonstrated the binding of melatonin to these transporters, and researchers have highlighted their appropriate binding structure to the receptor protein. Furthermore, after placing the transporters in the mitochondrial membrane, they were confirmed to promote melatonin uptake into this organ.

We speculate that the photoprotective effect and skin barrier construction function of melatonin and its metabolites directly or indirectly rely on the mitochondria. Additionally, the mitochondrial metabolism of melatonin is related to its direct or indirect (via metabolites) influence on mitochondrial function, ultimately producing various phenotypic effects. Considering the fact that melatonin has a 2.5 billion-year-old phylogenetic origin and functions, the epidermis, which lies between the external and internal environments, is an excellent model for assessing the connections between deleterious elements, local melatonin production, metabolic systems, and the mitochondria, which are modulators of visceral homeostasis and may have systemic results. Therefore, the epidermal melatonin system may characterize a feature/documentation of the conserved evolutionary function of this molecule and its metabolites as protectors against harmful external and internal elements. Melatonin and its metabolites will regulate the interaction of the mitochondria with skin cells to determine their survival or entry into precisely defined differentiation pathways, which are necessary for the barrier structure, or death via apoptosis pathways to inhibit carcinogenesis.

## 5. Conclusions

**Figure 4:**
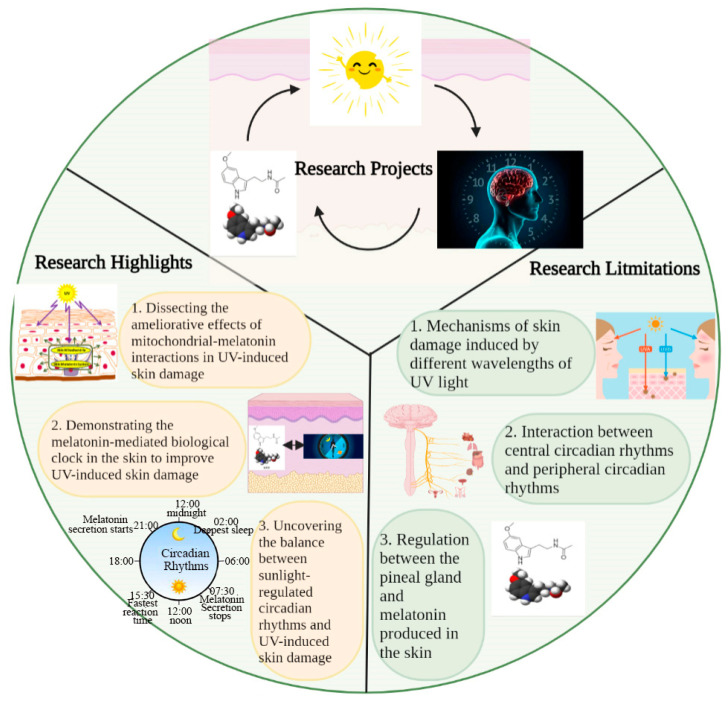
**Key elements, highlights, and limitations in this study.** This study discusses the role of interactions between sunlight (UV), circadian rhythm, and melatonin in regulating skin homeostasis. The highlights of this study are 1. dissecting the ameliorative effects of mitochondrial–melatonin interactions in UV-induced skin damage; 2. demonstrating the role of the melatonin-mediated biological clock in the skin in improving UV-induced skin damage; and 3. uncovering the balance between sunlight-regulated circadian rhythms and UV-induced skin damage. Some factors not discussed in this study include 1. mechanisms of skin damage induced by different wavelengths of UV light; 2. interaction between central circadian rhythms and peripheral circadian rhythms; and 3. relationship between melatonin secretion via the pineal gland and its production in the skin.

In this study, we discussed the mutual regulation and balances between sunlight, circadian rhythms, and the melatonin–mitochondrial axis in skin photoaging. In this study, we highlighted the balance between the role of sunlight in mediating circadian rhythms to maintain skin homeostasis and its role in mediating excessive ROS production to induce skin damage; in addition, we also revealed that melatonin itself mediates the biological clock of the skin to maintain skin health, in addition to regulating the central circadian rhythms for this purpose (Figure 4). These provide us with ideas to intervene in skin photoaging. However, there are also factors not addressed in this review, such as the mechanisms through which different wavelengths of UV light induce different degrees of skin damage, the regulation of circadian rhythms in the supraoptic nucleus and peripheral circadian rhythms, and the relationship between melatonin secretion via the pineal gland and melatonin production in the skin for the regulation of skin homeostasis. These are all issues to be explored in future studies, and we hope further investigation of these factors will also help to improve skin photoaging.

## Data Availability

Data will be made available on request due to privacy or ethical reason.

## Abbreviations

UVR: ultraviolet radiation; MMPs: matrix metalloproteinases; ROS: reactive oxygen species; ETC: electron transport chain; mtDNA: mitochondrial DNA; IL-1: interleukin-1; TNF-α: tumor necrosis factor-α; TGF-β: transforming growth factor-β; ECM: extracellular matrix; Tregs: regulatory T cells; Th1/Th2: T helper 1/2 cells; CPDs: cyclobutane pyrimidine dimers; SCN: suprachiasmatic nucleus; CLOCK: circadian locomotor output kaput; BMAL1: brain-and-muscle ARNT-like protein 1; PER: periods; CRY: cryptochromes; ROREs: retinoic-acid-related orphan receptor response elements; TEWL: transepidermal water loss; XPA: xeroderma pigmentosum group A; AQP3: aquaporin 3; C/EBPα: CCAAT-enhancer binding protein α; CXCL1: C-X-C motif ligand 1; AANAT: Arylalkylamine N-acetyltransferase; AFMK: N1-acetyl-N2-formyl-5-methoxykynuramine; AMK: N1-acetyl-5-methoxykynuramine; MPTP: mitochondrial permeability transition pore; GLUT1: glucose transporter 1; PEPT1/2: peptide transporters 1 and 2; SLC15A1/2: solute carrier family 15 members 1 and 2.

## References

[1] Yaar M., Gilchrest B.A., Goldsmith L.A., Katz S.I., Gilchrest B.A. (2012). Aging of skin. Fitzpatrick’s Dermatology in General Medicine.

[2] Vespa J., Armstrong D.M., Medina L. (2018). Demographic Turning Points for the United States: Population Projections for 2020 to 2060.

[3] Alzheimer’s Association (2018). 2018 Alzheimer’s disease facts and figures. Alzheimer’s Dement.

[4] Gasco-Buisson M.C., Farage M.A., Miller K.W., Maibach H.I. (2010). Key trends driving anti-aging skin care in 2009 and beyond. Textbook of Aging Skin.

[5] Deo M.S., Kerse N., Vandal A.C., Jarrett P. (2015). Dermatological disease in the older age group: A cross-sectional study in aged care facilities. BMJ Open.

[6] Blume-Peytavi U., Kottner J., Sterry W., Hodin M.W., Griffiths T.W., Watson R.E.B., Hay R.J., Griffiths C.E.M. (2016). Age-associated skin conditions and diseases: Current perspectives and future options. Gerontologist.

[7] Jabłońska-Trypuć A., Krętowski R., Kalinowska M., Świderski G., Cechowska-Pasko M., Lewandowski W. (2018). Possible mechanisms of the prevention of doxorubicin toxicity by cichoric acid-antioxidant nutrient. Nutrients.

[8] Slominski A.T., Slominski R.M., Raman C., Chen J.Y., Athar M., Elmets C. (2022). Neuroendocrine signaling in the skin with a special focus on the epidermal neuropeptides. Am. J. Physiol. Cell Physiol..

[9] Bocheva G., Slominski R.M., Slominski A.T. (2019). Neuroendocrine aspects of skin aging. Int. J. Mol. Sci..

[10] Vierkötter A., Hüls A., Yamamoto A., Stolz S., Krämer U., Matsui M.S., Morita A., Wang S., Li Z., Jin L. (2016). Extrinsic skin ageing in German, Chinese and Japanese women manifests differently in all three groups depending on ethnic background, age and anatomical site. J. Dermatol. Sci..

[11] Chien A.L., Qi J., Grandhi R., Kim N., César S.S.A., Harris-Tryon T., Jang M.S., Olowoyeye O., Kuhn D., Leung S. (2018). Effect of age, gender, and sun exposure on ethnic skin photoaging: Evidence gathered using a new photonumeric scale. J. Natl. Med. Assoc..

[12] Wohlrab J., Hilpert K., Wohlrab A. (2014). Characteristics of aging skin. Hautarzt.

[13] Orioli D., Dellambra E. (2018). Epigenetic regulation of skin cells in natural aging and premature aging diseases. Cells.

[14] Parisi A.V., Turner J. (2006). Variations in the short wavelength cut-off of the solar UV spectra. Photochem. Photobiol. Sci..

[15] Pustisek N., Situm M. (2011). UV-radiation, apoptosis and skin. Coll. Antropol..

[16] Zhang J., Bowden G.T. (2012). Activation of p38 MAP kinase and JNK pathways by UVA irradiation. Photochem. Photobiol. Sci..

[17] Slominski A.T., Zmijewski M.A., Plonka P.M., Szaflarski J.P., Paus R. (2018). How UV light touches the brain and endocrine system through skin, and why. Endocrinology.

[18] Cadet J., Douki T., Ravanat J.L. (2015). Oxidatively generated damage to cellular DNA by UVB and UVA radiation. Photochem. Photobiol..

[19] Slominski A.T., Zmijewski M.A., Skobowiat C., Zbytek B., Slominski R.M., Steketee J.D. (2012). Sensing the environment: Regulation of local and global homeostasis by the skin’s neuroendocrine system. Adv. Anat. Embryol. Cell Biol..

[20] Loughery J., Cox M., Smith L.M., Meek D.W. (2014). Critical role for p53-serine 15 phosphorylation in stimulating transactivation at p53-responsive promoters. Nucleic Acids Res..

[21] Wunderlich L., Paragh G., Wikonkál N.M., Bánhegyi G., Kárpáti S., Mandl J. (2008). UVB induces a biphasic response of HIF-1alpha in cultured human keratinocytes. Exp. Dermatol..

[22] Xian D., Lai R., Song J., Xiong X., Zhong J. (2019). Emerging perspective: Role of increased ROS and redox imbalance in skin carcinogenesis. Oxid. Med. Cell Longev..

[23] Bosch R., Philips N., Suárez-Pérez J.A., Juarranz A., Devmurari A., Chalensouk-Khaosaat J., González S. (2015). Mechanisms of photoaging and cutaneous photocarcinogenesis, and photoprotective strategies with phytochemicals. Antioxidants.

[24] Wooff Y., Si M.M., Aggio-Bruce R., Natoli R., Fernando N. (2019). IL-1 family members mediate cell death, inflammation and angiogenesis in retinal degenerative diseases. Front Immunol..

[25] Xu H., Wang C., Zhang H., Lv C., Wang Y. (2016). AstragalosideIV suppresses inflammatory mediator production in synoviocytes and collagen-induced arthritic rats. Mol. Med. Rep..

[26] Lee C.W., Lin Z.C., Hu S.C., Chiang Y.C., Hsu L.F., Lin Y.C., Lee I.T., Tsai M.H., Fang J.Y. (2016). Urban particulate matter down-regulates filaggrin via COX2 expression/PGE2 production leading to skin barrier dysfunction. Sci. Rep..

[27] Tsai M.H., Hsu L.F., Lee C.W., Chiang Y.C., Lee M.H., How J.M., Wu C.M., Huang C.L., Lee I.T. (2017). Resveratrol inhibits urban particulate matter-induced COX-2/PGE(2) release in human fibroblast-like synoviocytes via the inhibition of activation of NADPH oxidase/ROS/NF-κB. Int. J. Biochem. Cell Biol..

[28] Bridgeman B.B., Wang P., Ye B., Pelling J.C., Volpert O.V., Tong X. (2016). Inhibition of mTOR by apigenin in UVB-irradiated keratinocytes: A new implication of skin cancer prevention. Cell Signal.

[29] Zheng H., Zhang M., Luo H., Li H. (2019). Isoorientin alleviates UVB-induced skin injury by regulating mitochondrial ROS and cellular autophagy. Biochem. Biophys. Res. Commun..

[30] Derynck R., Akhurst R.J., Balmain A. (2001). TGF-beta signaling in tumor suppression and cancer progression. Nat. Genet..

[31] Kulkarni N.N., Adase C.A., Zhang L.J., Borkowski A.W., Li F., Sanford J.A., Coleman D.J., Aguilera C., Indra A.K., Gallo R.L. (2017). IL-1 receptor-knockout mice develop epidermal cysts and show an altered innate immune response after exposure to UVB radiation. J. Investig. Dermatol..

[32] Xu J., Feng Y., Song G., Gong Q., Yin L., Hu Y., Luo D., Yin Z. (2018). Tacrolimus reverses UVB irradiation-induced epidermal Langerhans cell reduction by inhibiting TNF-α secretion in keratinocytes via regulation of NF-κB/p65. Front. Pharmacol..

[33] Feng X., Yang M., Yang Z., Qian Q., Burns E.M., Min W. (2020). Abnormal expression of the co-stimulatory molecule B7-H3 in lichen simplex chronicus is associated with expansion of Langerhans cells. Clin. Exp. Dermatol..

[34] Chen N., Scarpa R., Zhang L., Seiberg M., Lin C.B. (2008). Nondenatured soy extracts reduce UVB-induced skin damage via multiple mechanisms. Photochem. Photobiol..

[35] Ling G., Ling E., Broides A., Poran Feldman H., Levy J., Garty B.Z., Nahum A. (2016). IL-12 receptor 1β deficiency with features of autoimmunity and photosensitivity. Autoimmunity.

[36] Behzadi P., Behzadi E., Ranjbar R. (2016). IL-12 family cytokines: General characteristics, pathogenic microorganisms, receptors, and signalling pathways. Acta Microbiol. Immunol. Hung..

[37] Zeng J., Luo S., Huang Y., Lu Q. (2017). Critical role of environmental factors in the pathogenesis of psoriasis. J. Dermatol..

[38] Sandu C., Dumas M., Malan A., Sambakhe D., Marteau C., Nizard C., Schnebert S., Perrier E., Challet E., Pévet P. (2012). Human skin keratinocytes, melanocytes, and fibroblasts contain distinct circadian clock machineries. Cell. Mol. Life Sci..

[39] Sarkar S., Gaddameedhi S. (2018). UV-B-induced erythema in human skin: The circadian clock is ticking. J. Investig. Dermatol..

[40] Buhr E.D., Takahashi J.S. (2013). Molecular components of the mammalian circadian clock. Handb. Exp. Pharmacol..

[41] Guillaumond F., Dardente H., Giguère V., Cermakian N. (2005). Differential control of Bmal1 circadian transcription by REV-ERB and ROR nuclear receptors. J. Biol. Rhythm..

[42] Yosipovitch G., Sackett-Lundeen L., Goon A., Yiong Huak C., Leok Goh C., Haus E. (2004). Circadian and ultradian (12 h) variations of skin blood flow and barrier function in non-irritated and irritated skin-effect of topical corticosteroids. J. Investig. Dermatol..

[43] Reinberg A.E., Soudant E., Koulbanis C., Bazin R., Nicolaï A., Mechkouri M., Touitou Y. (1995). Circadian dosing time dependency in the forearm skin penetration of methyl and hexyl nicotinate. Life Sci..

[44] Bruguerolle B., Giaufre E., Prat M. (1991). Temporal variations in transcutaneous passage of drugs: The example of lidocaine in children and in rats. Chronobiol. Int..

[45] Smolander J., Härmä M., Lindqvist A., Kolari P., Laitinen L.A. (1993). Circadian variation in peripheral blood flow in relation to core temperature at rest. Eur. J. Appl. Physiol. Occup. Physiol..

[46] Yosipovitch G., Xiong G.L., Haus E., Sackett-Lundeen L., Ashkenazi I., Maibach H.I. (1998). Time-dependent variations of the skin barrier function in humans: Transepidermal water loss, stratum corneum hydration, skin surface pH, and skin temperature. J. Investig. Dermatol..

[47] Lemmer B., Bruguerolle B. (1994). Chronopharmacokinetics. Are they clinically relevant?. Clin. Pharmacokinet..

[48] Al-Nuaimi Y., Hardman J.A., Bíró T., Haslam I.S., Philpott M.P., Tóth B.I., Farjo N., Farjo B., Baier G., Watson R.E.B. (2014). A meeting of two chronobiological systems: Circadian proteins Period1 and BMAL1 modulate the human hair cycle clock. J. Investig. Dermatol..

[49] Hardman J.A., Tobin D.J., Haslam I.S., Farjo N., Farjo B., Al-Nuaimi Y., Grimaldi B., Paus R. (2015). The peripheral clock regulates human pigmentation. J. Investig. Dermatol..

[50] Gupta M.A., Gupta A.K. (2013). Sleep-wake disorders and dermatology. Clin. Dermatol..

[51] Ozler M., Simsek K., Ozkan C., Akgul E.O., Topal T., Oter S., Korkmaz A. (2010). Comparison of the effect of topical and systemic melatonin administration on delayed wound healing in rats that underwent pinealectomy. Scand. J. Clin. Lab. Investig..

[52] Rogers N.L., Ferguson S., Amlaner C.J., Fuller P.M. (2009). Thermoregulation and sleep-wake behavior in humans. Basics of Sleep Guide.

[53] Leibowitz E., Seidman D.S., Laor A., Shapiro Y., Epstein Y. (1991). Are psoriatic patients at risk of heat intolerance?. Br. J. Dermatol..

[54] Li W.Q., Qureshi A.A., Schernhammer E.S., Han J. (2013). Rotating night-shift work and risk of psoriasis in US women. J. Investig. Dermatol..

[55] Ando N., Nakamura Y., Aoki R., Ishimaru K., Ogawa H., Okumura K., Shibata S., Shimada S., Nakao A. (2015). Circadian gene clock regulates psoriasis-like skin in ammation in mice. J. Investig. Dermatol..

[56] Le Fur I., Reinberg A., Lopez S., Morizot F., Mechkouri M., Tschachler E. (2001). Analysis of circadian and ultradian rhythms of skin surface properties of face and forearm of healthy women. J. Investig. Dermatol..

[57] Matsui M.S., Pelle E., Dong K., Pernodet N. (2016). Biological rhythms in the skin. Int. J. Mol. Sci..

[58] Park S., Kim K., Bae I.H., Lee S.H., Jung J., Lee T.R., Cho E.G. (2018). TIMP3 is a CLOCK-dependent diurnal gene that inhibits the expression of UVB-induced inflammatory cytokines in human keratinocytes. FASEB J..

[59] Park S., Lee E.S., Park N.H., Hwang K., Cho E.G. (2019). Circadian expression of TIMP3 is disrupted by UVB irradiation and recovered by green tea extracts. Int. J. Mol. Sci..

[60] Yeom M., Lee H., Shin S., Park D., Jung E. (2018). PER, a circadian clock component, mediates the suppression of MMP-1 expression in HaCaT keratinocytes by cAMP. Molecules.

[61] Slominski A., Wortsman J. (2000). Neuroendocrinology of the skin. Endocr. Rev..

[62] Slominski A., Fischer T.W., Zmijewski M.A., Wortsman J., Semak I., Zbytek B., Slominski R.M., Tobin D.J. (2005). On the role of melatonin in skin physiology and pathology. Endocrine.

[63] Slominski A. (2009). Neuroendocrine activity of the melanocyte. Exp. Dermatol..

[64] Shanahan T.L., Kronauer R.E., Duffy J.F., Williams G.H., Czeisler C.A. (1999). Melatonin rhythm observed throughout a three-cycle bright-light stimulus designed to reset the human circadian pacemaker. J. Biol. Rhythms.

[65] Vasey C., McBride J., Penta K. (2021). Circadian rhythm dysregulation and restoration: The role of melatonin. Nutrients.

[66] Janich P., Toufighi K., Solanas G., Luis N.M., Minkwitz S., Serrano L., Lehner B., Benitah S.A. (2013). Human epidermal stem cell function is regulated by circadian oscillations. Cell Stem Cell.

[67] Skobowiat C., Brożyna A.A., Janjetovic Z., Jeayeng S., Oak A.S.W., Kim T.K., Panich U., Reiter R.J., Slominski A.T. (2018). Melatonin and its derivatives counteract the ultraviolet B radiation-induced damage in human and porcine skin ex vivo. J. Pineal. Res..

[68] Kleszczyński K., Hardkop L.H., Fischer T.W. (2011). Differential effects of melatonin as a broad range UV-damage preventive dermato-endocrine regulator. Dermatoendocrinol.

[69] Slominski A.T., Zmijewski M.A., Semak I., Kim T.K., Janjetovic Z., Slominski R.M., Zmijewski J.W. (2017). Melatonin, mitochondria, and the skin. Cell Mol. Life Sci..

[70] Isapel N. (2018). New perspectives on the role of melatonin in human sleep, circadian rhythms and their regulation. Br. J. Pharmacol..

[71] Reiter R.J., Mayo J.C., Tan D.X., Sainz R.M., Alatorre-Jimenez M., Qin L. (2016). Melatonin as an antioxidant: Under promises but over delivers. J. Pineal. Res..

[72] Reiter R.J., Rosales-Corral S., Tan D.X., Jou M.J., Galano A., Xu B. (2017). Melatonin as a mitochondria-targeted antioxidant: One of evolution’s best ideas. Cell Mol. Life Sci..

[73] Hardeland R. (2017). Melatonin and the pathologies of weakened or dysregulated circadian oscillators. J. Pineal Res..

[74] Cardinali D.P., Hardeland R. (2017). Inflammaging, metabolic syndrome and melatonin: A call for treatment studies. Neuroendocrinology.

[75] Slominski A.T., Semak I., Fischer T.W., Kim T.K., Kleszczyński K., Hardeland R., Reiter R.J. (2017). Metabolism of melatonin in the skin: Why is it important?. Exp. Dermatol..

[76] Slominski A., Tobin D.J., Zmijewski M.A., Wortsman J., Paus R. (2008). Melatonin in the skin: Synthesis, metabolism and functions. Trends Endocrinol. Metab..

[77] Slominski R.M., Raman C., Chen J.Y., Slominski A.T. (2023). How cancer hijacks the body’s homeostasis through the neuroendocrine system. Trends Neurosci..

[78] Grando S.A., Pittelkow M.R., Schallreuter K.U. (2006). Adrenergic and cholinergic control in the biology of epidermis: Physiological and clinical significance. J. Investig. Dermatol..

[79] Schallreuter K.U., Bahadoran P., Picardo M., Slominski A., Elassiuty Y.E., Kemp E.H., Giachino C., Liu J.B., Luiten R.M., Lambe T. (2008). Vitiligo pathogenesis: Autoimmune disease, genetic defect, excessive reactive oxygen species, calcium imbalance, or what else?. Exp. Dermatol..

[80] Slominski A., Wortsman J., Tobin D.J. (2005). The cutaneous serotoninergic/melatoninergic system: Securing a place under the sun. FASEB J..

[81] Slominski A.T., Kleszczynski K., Semak I., Janjetovic Z., Zmijewski M.A., Kim T.K., Slominski R.M., Reiter R.J., Fischer T.W. (2014). Local melatoninergic system as the protector of skin integrity. Int. J. Mol. Sci..

[82] Zmijewski M.A., Sweatman T.W., Slominski A.T. (2009). The melatonin-producing system is fully functional in retinal pigment epithelium (ARPE-19). Mol. Cell. Endocrinol..

[83] Slominski A., Pisarchik A., Semak I., Sweatman T., Wortsman J., Szczesniewski A., Slugocki G., McNulty J., Kauser S., Tobin D.J. (2002). Serotoninergic and melatoninergic systems are fully expressed in human skin. FASEB J..

[84] Semak I., Korik E., Naumova M., Wortsman J., Slominski A. (2004). Serotonin metabolism in rat skin: Characterization by liquid chromatography-mass spectrometry. Arch. Biochem. Biophys..

[85] Slominski A., Pisarchik A., Semak I., Sweatman T., Szczesniewski A., Wortsman J. (2002). Serotoninergic system in hamster skin. J. Investig. Dermatol..

[86] Fischer T.W., Sweatman T.W., Semak I., Sayre R.M., Wortsman J., Slominski A. (2006). Constitutive and UV induced metabolism of melatonin in keratinocytes and cell-free systems. FASEB J..

[87] Kim T.K., Kleszczynski K., Janjetovic Z., Sweatman T., Lin Z., Li W., Reiter R.J., Fischer T.W., Slominski A.T. (2013). Metabolism of melatonin and biological activity of intermediates of melatoninergic pathway in human skin cells. FASEB J..

[88] Slominski A., Baker J., Rosano T.G., Guisti L.W., Ermak G., Grande M., Gaudet S.J. (1996). Metabolism of serotonin to N-acetylserotonin, melatonin, and 5-methoxytryptamine in hamster skin culture. J. Biol. Chem..

[89] Kobayashi H., Kromminga A., Dunlop T.W., Tychsen B., Conrad F., Suzuki N., Memezawa A., Bettermann A., Aiba S., Carlberg C. (2005). A role of melatonin in neuroectodermal-mesodermal interactions: The hair follicle synthesizes melatonin and expresses functional melatonin receptors. FASEB J..

[90] Kim T.K., Lin Z., Li W., Reiter R.J., Slominski A.T. (2015). N1-Acetyl-5-Methoxykynuramine (AMK) is produced in the human epidermis and shows antiproliferative effects. Endocrinology.

[91] Kim T.K., Lin Z., Tidwell W.J., Li W., Slominski A.T. (2015). Melatonin and its metabolites accumulate in the human epidermis in vivo and inhibit proliferation and tyrosinase activity in epidermal melanocytes in vitro. Mol. Cell Endocrinol..

[92] Pelle E., McCarthy J., Dong K., Layman D., Zamfifir R., Yarosh D.B., Pernodet N. (2012). Clock gene activity in keratinocytes measured with a per1-promoter construct. J. Investig. Dermatol..

[93] Hirayama J., Sahar S., Grimaldi B., Tamaru T., Takamatsu K., Nakahata Y., Sassone-Corsi P. (2007). CLOCK-mediated acetylation of BMAL1 controls circadian function. Nature.

[94] Nakahata Y., Kaluzova M., Grimaldi B., Sahar S., Hirayama J., Chen D., Guarente L.P., Sassone-Corsi P. (2008). The NAD+-dependent deacetylase SIRT1 modulates CLOCK-mediated chromatin remodeling and circadian control. Cell.

[95] Jung-Hynes B., Schmit T.L., Reagan-Shaw S.R., Siddiqui I.A., Mukhtar H., Ahmad N. (2011). Melatonin, a novel Sirt1 inhibitor, imparts antiproliferative effects against prostate cancer in vitro in culture and in vivo in TRAMP model. J. Pineal Res..

[96] Jung-Hynes B., Huang W., Reiter R.J., Ahmad N. (2010). Melatonin resynchronizes dysregulated circadian rhythm circuitry in human prostate cancer cells. J. Pineal Res..

[97] Reiter R.J., Tan D.X., Maldonado M.D. (2005). Melatonin as an antioxidant: Physiology versus pharmacology. J. Pineal Res..

[98] Fischer T.W., Scholz G., Knoll B., Hipler U.C., Elsner P. (2002). Melatonin suppresses reactive oxygen species in UV-irradiated leukocytes more than vitamin C and trolox. Skin Pharmacol. Appl. Skin. Physiol..

[99] Fischer T.W., Slominski A., Zmijewski M.A., Reiter R.J., Paus R. (2008). Melatonin as a major skin protectant: From free radical scavenging to DNA damage repair. Exp. Dermatol..

[100] Tan D.X., Manchester L.C., Qin L., Reiter R.J. (2016). Melatonin: A mitochondrial targeting molecule involving mitochondrial protection and dynamics. Int. J. Mol. Sci..

[101] Janjetovic Z., Jarrett S.G., Lee E.F., Duprey C., Reiter R.J., Slominski A.T. (2017). Melatonin and its metabolites protect human melanocytes against UVB-induced damage: Involvement of NRF2-mediated pathways. Sci. Rep..

[102] Kleszczynski K., Tukaj S., Kruse N., Zillikens D., Fischer T.W. (2013). Melatonin prevents ultraviolet radiation-induced alterations in plasma membrane potential and intracellular pH in human keratinocytes. J. Pineal Res..

[103] Sarti P., Magnifico M.C., Altieri F., Mastronicola D., Arese M. (2013). New evidence for cross talk between melatonin and mitochondria mediated by a circadian-compatible interaction with nitric oxide. Int. J. Mol. Sci..

[104] Janjetovic Z., Nahmias Z.P., Hanna S., Jarrett S.G., Kim T.K., Reiter R.J., Slominski A.T. (2014). Melatonin and its metabolites ameliorate ultraviolet B-induced damage in human epidermal keratinocytes. J. Pineal Res..

[105] Kleszczyński K., Zillikens D., Fischer T.W. (2016). Melatonin enhances mitochondrial ATP synthesis, reduces reactive oxygen species formation, and mediates translocation of the nuclear erythroid 2-related factor 2 resulting in activation of phase-2 anti-oxidant enzymes (γ-GCS, HO-1, NQO1) in ultraviolet radiation-treated normal human epidermal keratinocytes (NHEK). J. Pineal Res..

[106] Hevia D., Gonzalez-Menendez P., Quiros-Gonzalez I., Miar A., Rodriguez-Garcia A., Tan D.X., Reiter R.J., Mayo J.C., Sainz R.M. (2015). Melatonin uptake through glucose transporters: A new target for melatonin inhibition of cancer. J. Pineal Res..

[107] Mansouri A., Gaou I., de Kerguenec C., Amsellem S., Haouzi D., Berson A., Moreau A., Feldmann G., Letteron P., Pessayre D. (1999). An alcoholic binge causes massive degradation of hepatic mitochondrial DNA in mice. Gastroenterology.

[108] Martin M., Macias M., Escames G., Reiter R.J., Agapito M.T., Ortiz G.G., Acuna-Castroviejo D. (2000). Melatonin-induced increased activity of the respiratory chain complexes I and IV can prevent mitochondrial damage induced by ruthenium red in vivo. J. Pineal Res..

[109] Martin M., Macias M., Escames G., Leon J., Acuna-Castroviejo D. (2000). Melatonin but not vitamins C and E maintains glutathione homeostasis in t-butyl hydroperoxide-induced mitochondrial oxidative stress. FASEB J..

[110] Huo X., Wang C., Yu Z., Peng Y., Wang S., Feng S., Zhang S., Tian X., Sun C., Liu K. (2017). Human transporters, PEPT1/2, facilitate melatonin transportation into mitochondria of cancer cells: An implication of the therapeutic potential. J. Pineal Res..

